# A commentary on ‘Can adjuvant radiotherapy be omitted for oral cavity cancer patients who received neoadjuvant therapy and surgery? A retrospective cohort study’

**DOI:** 10.1097/JS9.0000000000000886

**Published:** 2023-11-16

**Authors:** Tongbin Liu, Detian Miao, Yu Zhang, Pengfei Jia, Peng Lin

**Affiliations:** a Department of Stomatology, Binzhou Medical University Hospital, Binzhou, Shandong; b QiLu Medical University, Zibo; c School of Stomatology, Qingdao University, Qingdao; d Department of Orthodontics, Jinan Stomatological Hospital, Jinan, Shandong, People’s Republic of China

*Dear Editor*,Oral cancer is one of the most common tumors worldwide, and for patients with locally advanced resectable oral squamous cell carcinoma (LAROSCC), adjuvant treatment after surgery usually includes radiotherapy^1^. Ju *et al*.^2^ conducted a retrospective cohort study to analyze patients with oral cancer who received neoadjuvant therapy and surgery. In certain circumstances, it may be possible to consider reducing the intensity of adjuvant therapy, such as omitting adjuvant radiotherapy.

The study included 192 LAROSCC patients who received neoadjuvant treatment and surgery at a medical center from 2008 to 2021. Patients were divided into two cohorts based on whether they received adjuvant radiotherapy: those who received adjuvant radiotherapy and those who did not. The study results showed that there was no significant difference in overall survival and local recurrence-free survival between the two cohorts. The 10-year survival rates of patients who received adjuvant radiotherapy and those who did not receive adjuvant radiotherapy were 58.9% and 44.1%, respectively, while the 10-year local recurrence-free survival rates were 55.4% and 48.2%, respectively. Further subgroup analysis showed that for clinical stage III patients, survival rates were not significantly different between the two cohorts, suggesting that omitting adjuvant radiotherapy may be a viable option in this specific patient subgroup.

The study also provides insight into other factors that may influence survival, including the pathological response of the primary tumor and nodal stage. However, adjuvant radiotherapy has not been shown to be an independent factor affecting survival. Although these results provide some preliminary evidence to support the omission of adjuvant radiotherapy in some patients with oral cancer, the limitations of this study also need to be noted. First, the sample size was limited and there was an imbalance in the number of patients between the cohorts who did and did not receive adjuvant radiotherapy, which may have introduced bias^3,4^. Second, since this is a retrospective study, there may be potential bias.

To verify this finding, larger and multicenter studies are needed. In addition, further study of indications and risk factors in different patient subgroups and more rigorous prospective studies and clinical trials are needed to determine the optimal treatment strategy. When designing future studies, the following aspects can be considered: First, conduct a large-scale multicenter study to validate the current findings and further explore indications and risk factors in different patient subgroups^5^. Secondly, when designing clinical trials, a de-escalation strategy can be used to make individualized decisions about whether to omit adjuvant radiotherapy based on the patient’s pathological response and other prognostic factors^6,7^. Finally, focus on the development of new adjuvant treatments to improve survival rates and quality of life for patients with oral cancer.

This study provides insight into whether adjuvant radiation therapy can be omitted in oral cancer patients undergoing neoadjuvant therapy and surgery. This issue has huge implications for clinical practice. This large retrospective cohort study provides preliminary evidence regarding the omission of adjuvant radiotherapy and lays the foundation for larger, multicenter studies. Possibilities and challenges for further research and potential applications of de-escalation therapy are also presented. This forward-looking thinking provides useful guidance and inspiration for the future development of this research field. In summary, this study, with its in-depth exploration and forward-looking advantages, provides preliminary evidence for the application of omitting adjuvant radiotherapy in the treatment of oral cancer and guides the development direction of future research in this field. Through further exploration and research, we can better guide clinical practice and optimize treatment strategies for oral cancer patients.

## Ethical approval

This manuscript is a comment. Do not need ethical approval.

## Consent

This manuscript is a comment. Do not need patient consent.

## Sources of funding

This manuscript is a comment without sources of funding.

## Author contribution

T.L. and D.M.: study concept or design, data collection, data analysis or interpretation, and writing the paper; Y.Z. and P.J.: data collection; P.L.: study concept or design, writing, and revising the paper.

## Conflicts of interest disclosure

This manuscript is a comment without conflicts of interest.

## Research registration unique identifying number (UIN)

This manuscript is a comment. Do not need UIN.

## Guarantor

Peng Lin.

## Data availability statement

This manuscript is a comment. Do not need a Data availability statement. However, all the data from the current study are publicly available.
